# Interactions Between Prolactin, Intracellular Signaling, and Possible Implications in the Contractility and Pathophysiology of Asthma

**DOI:** 10.3390/ijms26157332

**Published:** 2025-07-29

**Authors:** Eduardo Calixto, Juan C. Gomez-Verjan, Marco Cerbón, Valeria Rodríguez-Chávez, Bianca S. Romero-Martínez, María E. Martinez-Enriquez, Luis M. Montaño, Héctor Solís-Chagoyán, Arnoldo Aquino-Gálvez, Nadia A. Rivero-Segura, Georgina González-Ávila, Ana del Carmen Susunaga Notario, Gloria E. Pérez-Figueroa, Verónica Carbajal, Edgar Flores-Soto, Bettina Sommer

**Affiliations:** 1 Departamento de Neurobiología, Dirección de Investigación en Neurociencias, Instituto Nacional de Psiquiatría “Ramón de la Fuente Muñiz”, Mexico City 14370, CP, Mexico; ecalixto@inprf.gob.mx; 2 Dirección de Investigación, Instituto Nacional de Geriatría, Mexico City 10200, CP, Mexico; jverjan@inger.gob.mx (J.C.G.-V.); nrivero@inger.gob.mx (N.A.R.-S.); 3 Departamento de Biología, Facultad de Química, Universidad Nacional Autónoma de México, Mexico City 04510, CP, Mexico; mcerbon85@yahoo.com.mx (M.C.); valeria_rodriguez_chavez@hotmail.com (V.R.-C.); 4 Departamento de Farmacología, Facultad de Medicina, Universidad Nacional Autónoma de México, Mexico City 04510, CP, Mexico; biancasromero_@hotmail.com (B.S.R.-M.); emartinez@facmed.unam.mx (M.E.M.-E.); lmmr@unam.mx (L.M.M.); 5 Laboratorio de Neurobiología Cognitiva, Centro de Investigación en Ciencias Cognitivas, Universidad Autónoma del Estado de Morelos, Cuernavaca 62209, CP, Mexico; hector.solis@uaem.mx; 6 Laboratorio de Biología Molecular, Departamento de Fibrosis Pulmonar, Instituto Nacional de Enfermedades Respiratorias “Ismael Cosío Villegas”, Mexico City 14080, CP, Mexico; arnoldo.aquino@iner.gob.mx; 7 Laboratorio de Oncología Biomédica, Departamento de Enfermedades Crónico-Degenerativas, Instituto Nacional de Enfermedades Respiratorias “Ismael Cosio Villegas”, Mexico City 14080, CP, Mexico; ggonzalezavila@yahoo.com; 8 Secihti—ICAT Instituto de Ciencias Aplicadas y Tecnología, Universidad Nacional Autónoma de México, Mexico City 04510, CP, Mexico; ana.susunaga@icat.unam.mx; 9 Laboratorio de Inmunogenetica Bacteriana y Viral, Departamento de Microbiología y Parasitología, Facultad de Medicina, Universidad Nacional Autónoma de México, Mexico City 04510, CP, Mexico; gera.pfi3@gmail.com; 10 Departamento de Investigación en Hiperreactividad Bronquial, Instituto Nacional de Enfermedades Respiratorias “Ismael Cosío Villegas”, Mexico City 14080, CP, Mexico; estelacs@hotmail.com

**Keywords:** prolactin, PRL, asthma, contraction, hormonal regulation

## Abstract

Prolactin (PRL) is a hormone primarily associated with lactation, but it plays various roles in both men and women. PRL belongs to the family of peptide hormones, including placental lactogen and growth hormone. Interestingly, PRL is a pleiotropic hormone affecting several physiological and pathological conditions, including fertility. Moreover, several pathophysiological roles have been associated with this hormone, including those of the immune system, autoimmune disorders, asthma, and ageing. Additionally, PRL receptors are ubiquitously expressed in tissues, including the mammary gland, gonads, liver, kidney, adrenal gland, brain, heart, lungs, pituitary gland, uterus, skeletal muscle, skin blood cells, and immune system. Therefore, in the present paper, we cover the potential role that PRL may play in asthma by promoting inflammation and modulating immune responses. The detection of its receptor in lung tissue suggests a direct role in airway smooth muscle contractility through activation of signaling pathways such as JAK2-STAT5, MAPK/ERK1/2, and PI3K/Akt, as well as influencing ionic currents that regulate cell contraction, proliferation, and survival. In this sense, this review aims to explore the potential involvement of PRL in asthma pathophysiology by examining its interactions with intracellular signaling pathways and its possible impact on airway smooth muscle contractility and immune modulation.

## 1. Introduction

Asthma is a serious global health problem affecting approximately 300 million people of all ages around the world, and causing about 1000 deaths per day [1]. This ailment is a heterogeneous condition typically characterized by chronic airway inflammation, defined by a history of respiratory symptoms such as wheezing, shortness of breath, chest tightness, and cough that fluctuate over time and intensity, accompanied by variable limitations of expiratory air flow. According to the 2024 GINA [1], asthma can be classified in distinct clinical phenotypes, including allergic, nonallergic, late-onset, obesity-related, cough-predominant, and asthma with persistent airflow limitation. The best described variant is the allergic type, denominated atopic asthma, which results from an exacerbated Th2 response against antigens; exposure to them unleashes the onset of symptoms [2]. Unfortunately, it is a progressive illness, and severe cases often require treatment with inhaled corticosteroids [3]; in the worst scenario, symptoms turn refractory to this therapy [4]. Therefore, the search for alternative, reliable anti-inflammatory therapies for asthma treatment remains a hot topic. Asthmatic phenotypes differ in onset, inflammatory profiles, and treatment responses, highlighting the need for personalized approaches for diagnosis and management.

In addition, it has been proposed that the hypophysis–pituitary–adrenal (HPA) axis worsens asthma due to a physiological stress response [5], and unfortunately, this response orchestrated by the HPA axis still holds many unidentified features regarding this ailment. Conceivably, the management of asthma might expand substantially by increasing the knowledge of the role played by the HPA axis in asthma pathophysiology. In this sense, PRL, a hormone secreted by the pituitary gland, can modulate stress responses by inhibiting the HPA axis [6].

Growing evidence suggests that numerous hormonal factors play a role in lung development for optimal respiratory function, such as by influencing respiratory mechanics and inflammation. Some relevant factors involved in airway and lung illnesses are, for example, ghrelin, leptin, and glucagon-like peptide-1 (GLP-1) [7]; retinoids and cholecalciferol [8,9]; sex steroids [10]; and hormones such as insulin, prolactin [11], and glucagon [12], as well as growth factors like granulocyte/macrophage colony-stimulating factor (GM-CSF) [13].

Considering that the airway is subjected to neurohormonal modulation, new phenotypes have been proposed. Interestingly, sex hormones have been increasingly shown to play a substantial role in modulating smooth muscle contractility and to affect asthma development. In this sense, during periods of changes in female sex hormones, including puberty, menstruation, pregnancy, and menopause, these fluctuations have been associated with changes in asthma severity [14,15,16]. Pregnancy in asthmatic women becomes a particular concern, since during gestation, one-third of pregnant women suffer worsening asthma symptoms and one-third improve, while the remaining one-third show no change [16,17,18]. Interestingly, in a study including 308 asthmatic pregnant women, Stevens et al. did not find any improvement in any of these patients, reporting either a worsening of symptoms (40.5%) or no change (59.5%) [19]. These findings indicate that the hormonal fluctuations present during pregnancy strongly influence asthma, and this could be of importance during other periods of change, such as menopause. Recently, a so-called “late-onset phenotype” has been distinguished affecting older obese women [20,21] and otherwise healthy women during menopause [21], indicating that female sex hormones or their absence may play a role in airway hyperresponsiveness and asthma development [17,20,21]. In this sense, although little is known of the role that prolactin (PRL) could potentially play in asthma, there are clear indications that, since the airway is subjected to hormonal control, PRL could also participate in the pathophysiology and merits further research.

Nowadays, PRL, traditionally associated with lactation, is recognized for its immunomodulatory effects [22,23]. It is currently recognized that PRL is a pleiotropic hormone that participates in more than 300 physiological functions, including reproduction, metabolism, immune response, and brain processes. PRL has been reported to cross the blood–brain barrier and exert its effects on various regions of the central nervous system. Furthermore, recent studies have demonstrated its involvement in immunological mechanisms, suggesting that it plays a relevant role in inflammation [11,24]. On the other hand, PRL has been associated with many autoimmune diseases, such as systemic lupus erythematosus (SLE) [25,26,27], rheumatoid arthritis (RA) [25], and multiple sclerosis (MS) [25]. Furthermore, it has been observed that, while aging, PRL levels fluctuate, and that both increases and decreases in these concentrations have been linked to neurodegenerative diseases (including Huntington’s, multiple sclerosis, Alzheimer’s, and Parkinson’s) [28,29].

**Figure 1:**
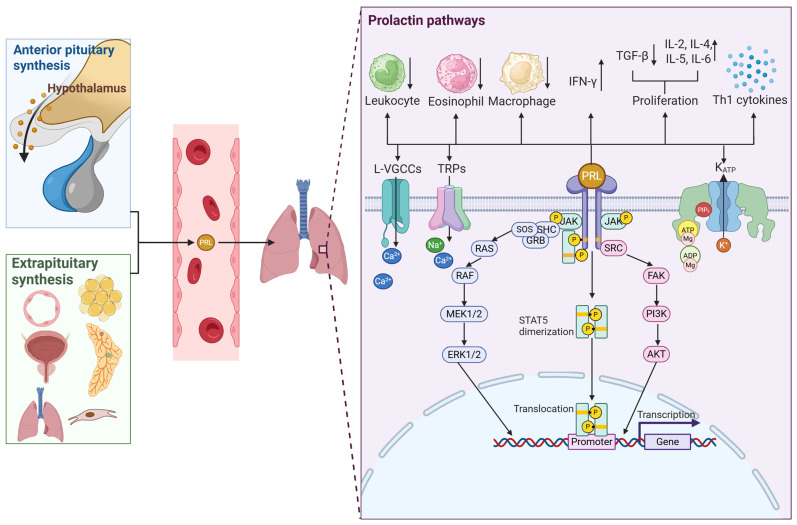
Proposed effects of prolactin in the lungs. Prolactin (PRL) can be synthesized either in the pituitary gland or in extrapituitary tissues, such as endothelial cells, adipocytes, the prostate, mammary glands, lungs, fibroblasts, etc. Prolactin then can be released into the blood stream to reach its target organ. In the lung, prolactin can exert immunomodulatory effects, activity over ion channels, and regulation of intracellular processes mediated by the activation of the MEK1/2/ERK1/2, JAK/STAT5, and PI3K/AKT pathways.

However, in asthma, PRL might be involved in promoting inflammation and modulating immune cell activity [11,30]. Additionally, the presence of its PRL receptor (PRLR) has been observed in lung tissue in animal models [31], suggesting that this hormone could be directly involved in airway smooth muscle contraction, because PRLR stimulation activates the JAK2-STAT5 signaling pathway, which participates in processes such as contraction [32], proliferation [33,34,35], differentiation [34,35,36], and cell survival [34,35,36,37]. It has also been reported that PRL activates other signaling pathways, such as MAPK/ERK1/2 and PI3K/Akt, expanding its impact on diverse cellular functions (Figure 1) [35,38,39,40,41].

Interestingly, augmented plasma PRL concentration modulates ionic currents in multiple tissues from different species. For example, it modulates ATP-sensitive potassium channels to provoke an analgesic response in mice, significantly increasing the active transport of Ca^2+^ in duodenal enterocytes, an effect that is completely abolished by blocking L-type calcium channels with nifedipine or by inhibiting the main Ca^2+^ elimination systems in the basolateral membrane, e.g., plasma membrane Ca^2+^-ATPase (PMCA) and the Na^+^/Ca^2+^ exchanger (NCX) (Figure 1) [42]. Meanwhile, in sensory neurons of female rats, PRL potentiates the activity of acid-sensitive ion channels in primary sensory neurons [43]. It has also been shown that this hormone induces small electrical currents in neurons through TRP-like calcium channels and facilitates Ca^2+^ entry via voltage-gated L-type calcium channels. Furthermore, it has been suggested that its rapid responses in neuronal cells are mediated by the short isoform of the PRLR, activating intracellular signaling pathways, such as PI3-kinase and PKC [40]. It stimulates sodium and chloride transport in renal epithelial cells (A6) by activating the epithelial sodium channel (ENaC) and a chloride channel (ClC4)-type anion channel. This effect depends on the cAMP/PKA signaling pathway, since its inhibition blocks the response. PRL increases both the number and the probability of ENaC opening, thereby promoting amiloride-sensitive and -insensitive trans-epithelial currents [44]. Finally, PRL was also observed to activate ENaC and ClC channels in A6 renal epithelial cells via the cAMP/PKA-dependent signaling pathway [44].

Despite evidence that PRL can have effects in the lungs and airways [30,45], its specific role in asthma pathophysiology remains poorly understood and largely unexplored in clinical settings. Conceivably, it might possess a potentially important role in the pathophysiology of asthma, which may be particularly relevant for women during pregnancy and lactation. The objective of this study is to analyze the possible role of prolactin in the modulation of airway smooth muscle excitation–contraction coupling.

## 2. Prolactin

Currently, PRL interests the scientific community due to its multiple functions in the organism [37,46,47]. PRL was discovered in the 1930s by biologist Oscar Riddle through studies in pigeons where an unidentified anterior pituitary hormone controlled the production of “crop-milk” [48]. Interestingly, at first, it was only recognized as a factor that controls milk production and secretion [46,48,49,50]. However, it was not until the 1970s that Dr. Henry Friesen et al. succeeded in purifying and characterizing human prolactin from pituitary extracts [51]. Nevertheless, it is now recognized as a pleiotropic hormone, with over 300 functions in numerous tissues [47,52,53].

PRL belongs to the family of peptide hormones that also includes placental lactogen and growth hormone, making up the family of somatolactogens, also known as class I helical cytokines [50,54]; these hormones are characterized by having a tertiary structure, composed of four antiparallel α helices [35,47]. PRL is synthesized in the anterior lobe of the pituitary gland by specialized cells called lactotrophs [39,47,55,56]. However, it has also been reported that it is synthesized extrapituitarily in other tissues, such as lymphocytes, skin fibroblasts, prostate cells, endothelial cells, adipose tissue cells, mammary gland, ovaries, and decidua, and in the brain (Figure 1) [24,35,37,57,58].

Regarding its structural characteristics, it has been reported that PRL is encoded by a single gene (PRL), whose size is 10 kb. This gene is composed of five exons and four introns in most species, including mammals, fish, and birds [35,37,39,47,59,60]. Furthermore, it has been reported that the transcriptional regulation of pituitary and extrapituitary PRL expression is under the control of two independent promoter regions: the first is the proximal promoter region that modulates pituitary expression of PRL, and the second is a distal promoter region that promotes extrapituitary PRL expression [33,35,37,47].

However, at the molecular level, its expression and regulation are tissue-specific because several isoforms of PRL have been described. This is the result from alternative splicing, proteolytic cleavage, or post-translational modifications (such as phosphorylation, glycosylation, and deamidation) and the association with other circulating proteins. It is important to note that these modifications can modify the biological activity of PRL in the organism [35,39,47,61].

**Figure 2:**
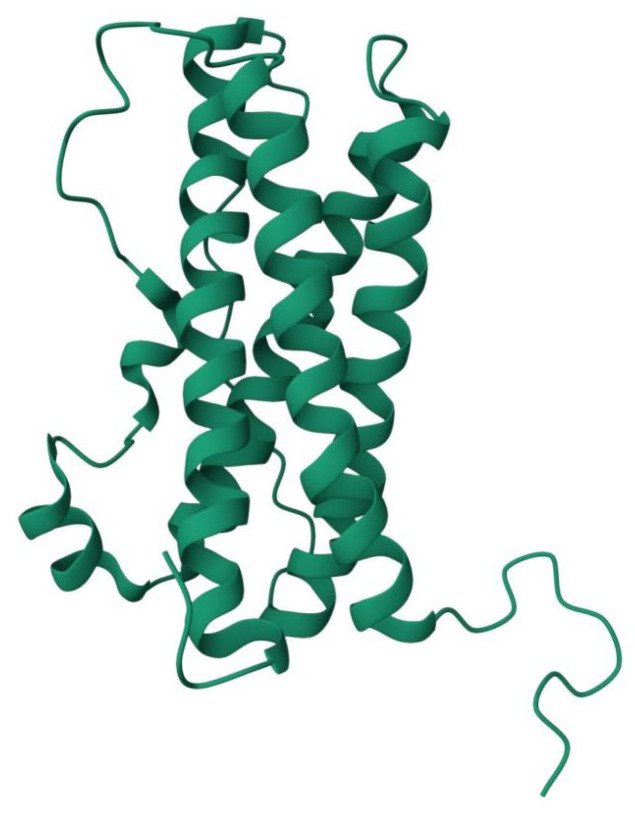
PRL structure (ID: 1RW5) obtained from the Protein Databank (https://www.rcsb.org/, accessed on 11 July 2025).

In addition, it has been reported that PRL is composed of 199 amino acid residues with a weight of 23 kDa. This form is known as monomeric PRL (little PRL), and it has three disulfide bonds present in similar places in rodents and humans (Figure 2) [33,37,39,52].

**Figure 3:**
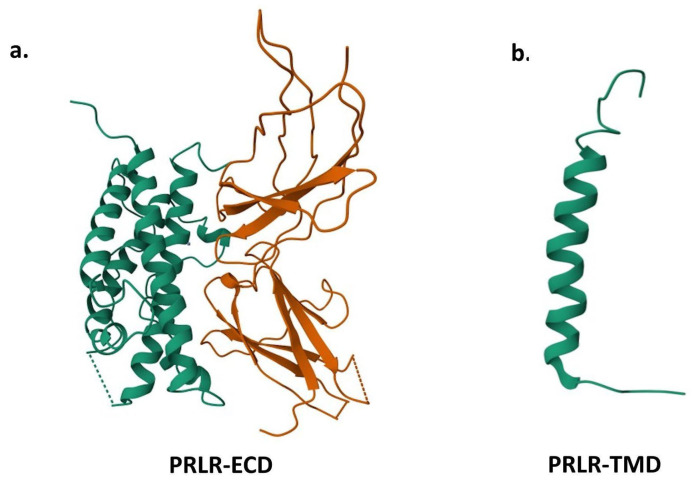
PRLR domain structures obtained from the Protein Databank (https://www.rcsb.org/, accessed on 11 July 2025). (**a**) PRLR-ECD (ID: 1BP3) [74], (**b**) PRLR-TDM (ID: 2N7I) [75].

Likewise, it has been reported that PRL can form dimers, polymers, and aggregates [35,47,61,62]. Some examples of the modifications that PRL can have are as follows: a high molecular mass such as that of the “big PRL” and the “big-big PRL” (also known as macroPRL), of approximately 100 kDa, which has been reported in the blood tissue [37,47,62,63]. Nevertheless, macroPRLs show lower biological activity, and it is suggested that they participate in the storage, modification, and release of PRL [35,47,64]. On the other hand, there are PRLs with low molecular weight, which can be 14 kDa, 16 kDa and 22 kDa; these forms are generated from the proteolytic cleavage of the 23 kDa pituitary PRL [33,46,50,61,65]. An example of a molecular fragment derived from the proteolytic processing of PRL is vasoinhibin, generated by the action of proteases such as cathepsin D [61,65,66]. Finally, to exert its biological action, PRL requires interaction with its receptors, which are expressed in various tissues and organs (Figure 3) [35,36,60,67].

## 3. Prolactin Receptors

As previously reported, the actions of PRL are initiated when this hormone binds to a homodimer of the PRLR, forming a heterotrimeric complex [36,37,38]. Therefore, it is essential to describe the characteristics of PRLRs, which are ubiquitously expressed in organisms [36,38,40,60] and their tissues, including the mammary gland, gonads, liver, kidney, adrenal gland, brain, heart, lungs, pituitary gland, uterus, skeletal muscle, skin, blood cells, and immune system [36,47,50,68,69,70,71]. It has been reported that PRLRs are transmembrane proteins and belong to class I of the cytokine receptor superfamily. Interestingly, they lack intrinsic tyrosine kinase activity and can be phosphorylated by cytoplasmic proteins [36,37,40,60]. It should be noted that this family also includes receptors for GH (GHR), leptin (LEPR), leukemia inhibiting factor (FIL), and erythropoietin (EPO), to mention a few [35,37,50].

PRLRs are composed of three domains: extracellular, transmembrane, and intracellular [40,50,60]. In fact, Araya-Secchi, R et al. report that the full human PRLR structure is ∼345 Å on the vertical axis from N to the C terminus, and the extracellular domain (ECD) constitutes ∼20%, the transmembrane domain (TMD) occupies ∼10%, and the intracellular domain (ICD) constitutes ∼70% [72,73]. In this sense, the ECD (Figure 3a) includes two regions, called S1 and S2 (or D1 and D2), which form together the ligand binding site and are identical between species. The only difference is the intracellular cytoplasmic domain, which varies in length [35,37,40,50,60]. Modifications in this domain are indispensable to recognize the different PRLRs, because, like PRL, alternative mRNA splicing can occur in the PRLR. Additionally, the PRLR-TDM (Figure 3b) is characterized as an α-helix that seems to be identical between species. For a better understanding of the PRLR-TDM, we recommend Araya-Secchi, R.

Currently, three PRLR isoforms have been described in rodents and five in humans. In both species the long, the intermediate, and the short isoforms have been extensively studied [36,40,50,64]. The union of two PRLR monomers of the same isoform leads to the formation of homodimers or of heterodimers when different isoforms join [50,60]. Interestingly, a soluble isoform has been described only in humans [34,37,64,76,77]. This soluble isoform has been characterized in human breast cancer cell lines and seemingly functions as a regulatory mechanism for the bioavailability and signaling of extrapituitary PRL, which is why it is also known as “PRL binding protein” in the extracellular domain [76,77,78].

On the other hand, PRLRs have mainly been related to the activation of the JAK2-STAT5 signaling pathway [33,34,35,36,37]. This signaling pathway is activated in response to cytokines, growth factors, and PRL. Furthermore, it is implicated in multiple functions in secretory mammary epithelial cells, which include specifying, proliferating, differentiating, and surviving [33,34,60,71,78]. Also, it is essential to mention that the discovery of this signaling cascade was a significant advance in the understanding of the actions of PRL in the body [33,35,37,38,78]. Nevertheless, it has also been described that PRL can induce the activation of two other pathways, which are the MAPK/ERK1/2 pathway and the P13K/Akt pathway [35,38,39,40,41]. Activation of all these pathways can influence the various functions described for PRL.

## 4. Prolactin Functions

The canonical PRL function is to promote lactation; during this stage, the secretion of PRL is regulated by a delicate mechanism of endogenous factors such as PRL-releasing factors (PRFs), as well as thyrotropin-releasing hormone (THR), and by external factors such as photoperiod, food availability, and physical processes associated with breast suction that stimulate its secretion. High concentrations of PRL induce the release of PRL inhibitory factors (PIFs) like gamma-aminobutyric acid (GABA), dihydroxyphenylalanine (DOPA), and somatostatin [35,50,55,79,80]. Currently, it is known that PRL also regulates a large number of physiological processes, and, therefore, it is considered a pleiotropic hormone with more than 300 described functions divided into categories like reproduction, brain processes, immune responses, angiogenesis maternal behavior, growth, metabolism, and osmoregulation [24,38,47,52,58,65,66,78,81].

It has been reported that PRL can cross the blood–brain barrier [46,55,58,82], while its mRNA has been reported in several brain areas, such as the olfactory bulb, the corpus callosum, the choroid plexuses, the amygdala, the hypothalamus, the thalamus, the cerebral cortex, and the hippocampus [33,35,36,38,39,46,60]. However, data on PRL expression in the brain remains limited and controversial. Since no conclusive studies demonstrate PRL secretion in the brain, it is proposed that all PRL effects arise from a pituitary source. However, a recent transcriptomic survey by Cabrera-Reyes et al., suggests that PRL exerts some effects in the hippocampus [83]. Nevertheless, PRL actions on the brain depend on factors such as age, sex, and the reproductive status of the species [34,35,37,38,40,41,71]. Finally, it is relevant to mention that PRL has been reported to participate in many brain functions, including maternal behavior, memory, energy balance, food intake, sleep, anxiety, neurogenesis, and neuroprotection [40,41,52,67,69,84,85,86,87,88,89], the latter being a field of interest for the scientific community.

## 5. Prolactin and Its Interactions

**Figure 4:**
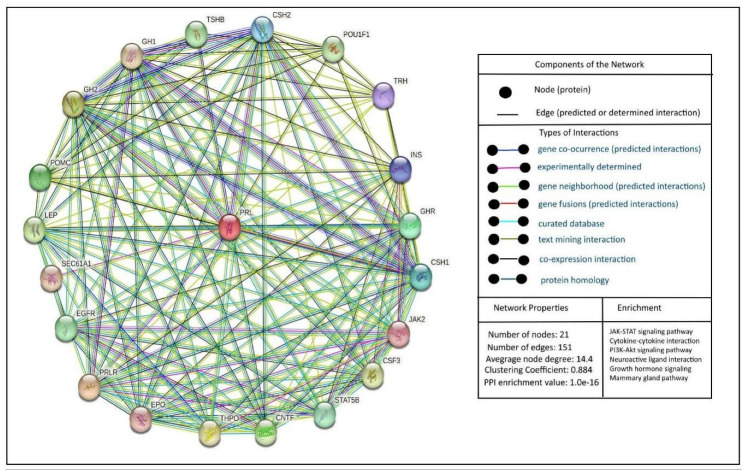
Top 20 PRL-PPI interactions. The figure indicates the top 20 protein-protein interactions of PRL according to the STRING database (https://string-db.org/, accessed on 1 March 2025) from *Homo sapiens*. On the right side of the figure, we can see the types of interactions, network properties, and enrichment analysis of the main pathways involved in the PRL network.

When discussing PRL, it is essential to focus on the interactions between its receptor and its downstream signaling pathways. It is necessary to indicate that PRLR’s interactions are complex and involve multiple signaling pathways; among them, the JAK/STAT pathway (Figure 4).

It is essential to mention that PRL is a pleiotropic hormone that can affect several physiological and pathological functions, including fertility. Moreover, PRLRs are widely expressed in several tissues, including brain regions and reproductive organs. Thus, PRLRs may exert prolactin’s functions upon activation through several signaling pathways, as seen in Figure 1.

## 6. Prolactin and the Immune System

Interestingly, the genes that encode PRL are located in the short arm of chromosome 6, in proximity to the HLA-DRB1 gene that is associated with autoimmune diseases, especially SLE [90,91]. Furthermore, prolactin has been shown to act as an immunomodulator. In splenic B cells from female Balb/c mice, prolactin treatment for 4 weeks led to a decrease in the BCR-mediated apoptosis of the T1 B cell subset, which was associated with an upregulation of the anti-apoptotic gene INF-γRII and downregulation of the pro-apoptotic *Trp63* gene [92]. Additionally, the state or hyperprolactinemia also dysregulates receptor editing by causing the co-expression of more than one light chain in the cell’s surface; thus, the cell can escape clonal deletion and generate autoreactivity, as well as modify the level of B cell anergy by lowering the BCR-mediated activation threshold [92]. In B cell hybridomas, PRL, in a dose-dependent manner, increases proliferation induced by IL-4, IL-5, and IL-6 and decreases the downregulation in proliferation induced by TGF-β (Figure 1) [93,94].

In T cells, PRL treatment (2–200 ng/mL) augmented proliferation following IL-2 and phytohemagglutinin stimulation without modifying the subsets [95]. PRL also modulates dendritic cell (DC) differentiation and maturation. At physiological levels (10–20 ng/mL), PRL acts synergically with GM-CSF to inhibit DC maturation, comparable to the effect induced by IL-4, while supraphysiological concentrations (80 ng/mL) have the opposite effect [91,96]. PRL modulates IFN-γ production through the JAK/Stat/IRF-1 pathway, in addition to the modulation of DC cells, thus leading to an inflammatory response that could be implicated in SLE patients [91,97,98,99,100]. Furthermore, in murine spleen CD1c-positive dendritic cells (SDCs), 24 hr PRL treatment increased viability, stimulatory capacity, and CD40 and MHC-11 expression, while decreasing the levels of CD54, NF-κBp65, and endocytosis [101,102].

As for cytokine-mediated PRL production, it can both increase and decrease. While IL-1, IL-2, and IL-6 have been shown to stimulate PRL secretion, IFN-γ inhibits, and TNF-α has both stimulatory and inhibitory effects [94,103,104,105,106]. PRL has also been shown to enhance Th1 type cytokines in vivo and in vitro [94,96,107,108], increase the release of IL-12 [107] and IL-1 in mouse peritoneal macrophages [109], and increment IL-6 production via PRL-mediated IL-1 activity (Figure 1) [110].

## 7. Prolactin and Autoimmune Diseases

Autoimmune diseases are influenced by hormonal regulation, with their higher prevalence in females suggesting a significant role for sex hormones in the underlying pathophysiology. Various diseases from this group have been associated with elevated circulating levels of PRL [90,91,111,112,113]. SLE activity has been positively associated with increased serum levels of PRL [91,111,114]. Treatment with bromocriptine, a dopamine receptor agonist that selectively inhibits prolactin secretion, has been demonstrated to effectively suppress the disease in NZB/NZW (B/W) F1 mice, (a SLE-like animal model), where lower anti-DNA antibodies and circulating IgG levels were observed, as well as a longer lifespan [115]. Similarly, in SLE patients, bromocriptine treatment has been associated with less disease severity and lower anti-dsDNA. Noticeably, the patients presented flare-ups when the therapy was discontinued [91,116].

Hyperprolactinemia was found to be associated with antiphospholipid syndrome, especially with reproductive failure presented in the disease [112]. Furthermore, in a case report, hyperprolactinemia was found in a patient with multiple autoimmune diseases (Jaccoud’s arthropathy, urticarial vasculitis, systemic lupus erythematosus, and Sjögren’s syndrome) [90], correlating with the 20% of patients with SLE that present increased serum levels of prolactin [90,113].

## 8. Prolactin in Asthma

PRL, commonly recognized for its role in lactation, has gained attention as an immunomodulator and participant in the pathogenesis of immune diseases. Although little is known, emerging evidence suggests that PRL could be involved in asthma by modulating immune cell function and promoting a proinflammatory state. Interestingly, in one study, female rats were divided into three groups: virgins with no lung injury (n group), ovalbumin (OVA)-sensitized virgins (V group), and OVA-sensitized lactating females (L group). OVA sensitization is a useful animal model for allergic asthma. The L group had a lower bronchoalveolar lavage (BAL) leukocyte count and eosinophil and macrophage count compared with the V group; meanwhile, BAL interferon-γ concentrations were increased and corticosterone levels were decreased. Interestingly, norepinephrine levels were higher in the L group when compared to the N and V groups. These results indicate that lactation hampered the OVA-sensitized females in developing a proper inflammatory response, and, although not clearly stated in this research, prolactin might have contributed to this effect (Figure 1) [45].

In contrast, another study, involving 86 children with mild asthma treated either with sublingual immunotherapy (SLIT) or given a placebo for 6 months, showed that lower symptoms might be related to the reduction in PRL and the consequent lower activation of T lymphocytes. At the end of the study, the treated group reported a significant improvement in asthma and rhinitis symptoms and lower serum levels of eosinophil cationic protein (ECP), IL-13, PRL, and adrenocorticotropic hormone (ACTH). The reduction in the Th2 cytokine response and symptomology could be linked to an immunomodulatory effect of PRL suppressed by the SLIT treatment [30].

In women with perimenstrual asthma (PMA), serum prolactin levels tend to increase during both the luteal and follicular phases compared to asthmatic patients without PMA and healthy subjects [117]. Although a significant difference has not been observed, this does indicate a possible relation between prolactin and asthma symptomology that warrants further study. Recently, in OVA-sensitized asthmatic mice, treatment with the aqueous extract of Herba Houttuyniae reduced airway hyperresponsiveness (AHR; a hallmark of asthmatic airways) to methacholine. It was established that the therapeutic effect observed was induced by six metabolites and the prime targeted candidate to suppress asthma-related genes was related to the prolactin signaling pathway (Table 1) [118].

## 9. Prolactin and Aging

Aging is characterized by a subtle decline in all biological systems function, including the endocrine ensembles, particularly at the central regulator of endocrine hypothalamic-pituitary units. In this sense, it has been reported that during aging, prolactin secretion decreases by about 40% after menopause but declines less in older men. Since it has been reported that PRL-R is downregulated in the aged retina, it has been suggested that PRL signaling is impaired, leading to retinal dysfunction, suggesting that PRL is required for the homeostasis of aged retina [123]. On the other hand, PRL dysregulation has been associated with neurodegenerative diseases, including Huntington’s, multiple sclerosis [28], Alzheimer’s (AD), and Parkinson’s diseases (PD) [29]. In the early stages of AD, PRL levels are significantly increased [124], probably since individuals with AD show significant alterations in the tubero-infundibular pathway involved in the regulation of PRL secretion [125]; however, the levels decrease in the late phase of the disease. Similarly, in older adults with PD, serum levels of PRL are increased when compared to age-matched controls [126]. In this sense, the rise of PRL in older men has been reported to influence cognition, mood, and quality of life [127]. One of the primary markers of aging is the “biological clock”, based on DNA methylation, and an acceleration in biological age is significantly associated with the risk of developing breast cancer [128]. With this in mind, higher levels of PRL are moderately associated with an increased risk of breast cancer [129]. Since PRL fluctuations are present in menopause, this could affect cancer risk. Finally, PRL has been recognized to influence bone density [130]; in particular, postmenopausal women with hyperprolactinemia show multiple effects on bone metabolism, affecting both bone mass and density, which in turn is associated with a high risk of osteoporosis [131]. During menopause, asthma symptomatology might develop; this phenotype has been termed “late-onset asthma” and is associated with fluctuations in hormone levels [21]. Sex hormones are known modulators of airway hyperresponsiveness and inflammation, and their reduction can influence asthma development and severity [17,21]. Prolactin, a well-known pleiotropic hormone with immunomodulatory effects, could enhance pro-inflammatory cytokine production [94,96,107,108,110] and airway inflammation [45]. Because prolactin levels are also altered during menopause, they may also contribute to asthma pathophysiology.

Nonetheless, the consequences of PRL concentrations while aging remain controversial since low or high levels lead to the impairment of multiple organs, contributing to the organism’s decline. In this context, it is important to highlight that understanding the fluctuation of PRL levels during aging is imperative to design more effective treatments that target the most common age-related diseases.

## 10. Conclusions

In addition to its conventional role in lactation, there is ample evidence supporting the role of prolactin in modulating the function of the immune system and the ion transport through the cell membrane, either through direct or indirect mechanisms. These facts suggest the participation of prolactin in the pathogenesis of asthma by impacting the immune function and promoting airway smooth muscle contraction through intracellular signaling pathways.

## Figures and Tables

**Table 1:** Clinical and physiological conditions associated with changes in prolactin levels and their possible relationship with asthma.

Aspect	Prolactin/Asthma Relationship	Proposed Mechanism/Evidence	Reference
Drug-induced hyperprolactinemia	↓ Allergic lung inflammation	Inhibits D2 receptors → ↑ Prolactin → Modulates leukocytes, mucus, and cytokines (IL-4, IL-6, IL-10, TNF-α)	[11]
Pregnancy and Breastfeeding	↑ Prolactin during breastfeeding = possible protection	Lactating females → ↑ plasma prolactin Modulation of the inflammatory response Stimulates B lymphopoiesis and the secretion of IL-1, IL-6, and TNF-α Mast cell involvement Promotes bronchodilation via the β2 pathway	[45,119]
Premenstrual phase	Possible indirect effect via hormonal disruption	Hyperprolactinemia → Anovulation → ↓ Progesterone/estrogens → Indirect impact on asthma exacerbations	[120]
Stress	↑ Prolactin associated with stress → potential asthma exacerbation	Activation of the HPA and sympathetic axis → Inflammatory modulation; Hyperprolactinemia as an immunological mediator	[121,122]
Environmental factors	Possible proinflammatory role of prolactin	↑ Cytokines due to pollution → ↑ Prolactin → Modulates respiratory immune response	[119]

## Data Availability

Not applicable.

## Abbreviations

The following abbreviations are used in this manuscript:

PRL	Prolactin
GINA	Global Initiative for Asthma
HPA	Hypophysis–pituitary–adrenal
SLE	Systemic lupus erythematosus
RA	Rheumatoid arthritis
MS	Multiple sclerosis
PRLR	Prolactin receptor
PMCA	Plasma membrane Ca^2+^-ATPase
NCX	Na^+^/Ca^2+^ exchanger
ENaC	Epithelial sodium channel
ClC4	Chloride channel
L-VGCCs	L-type voltage-gated calcium channels
TRPs	Transient receptor potential
K_ATP_	ATP-sensitive potassium channels
FIL	Leukemia inhibiting factor
EPO	Erythropoietin
PRFs	PRL-releasing factors
PIFs	PRL inhibitory factors
GABA	Gamma-aminobutyric acid
DOPA	Dihydroxyphenylalanine
DC	Dendritic cell
GM-CSF	Granulocyte/macrophage colony-stimulating factor
SDCs	CD1c-positive dendritic cells
OVA	Ovalbumin
SLIT	Sublingual immunotherapy
ECP	Eosinophil cationic protein
ACTH	Adrenocorticotropic hormone
PMA	Perimenstrual asthma
AHR	Airway hyperresponsiveness
